# An updated look at the use of silver diamine fluoride in U.S. dental schools' predoctoral curriculum—a survey

**DOI:** 10.3389/fdmed.2024.1466962

**Published:** 2025-01-14

**Authors:** Benas Jakubauskas, Sarah Pagni, Andrea G. Ferreira Zandona

**Affiliations:** ^1^ Rollins Family Dental Center, Round Lake Beach, IL, United States; ^2^ Department of Public Health and Community Service, Tufts University School of Dental Medicine, Boston, MA, United States; ^3^ Department of Restorative and Prosthetic Sciences, The Ohio State University College of Dentistry, Columbus, OH, United States

**Keywords:** cariology, dental caries, dental curriculum, dental education, silver diamine fluoride

## Abstract

This survey aimed to update Silver Diamine Fluoride (SDF) use/teaching in U.S. predoctoral dental education programs in comparison to a 2016 survey, considering the COVID-19 pandemic. An online survey via email was sent to all accredited U.S. predoctoral dental education programs (*n* = 68) in January 2022. A total of 39 schools (57% response rate) responded to the survey, and all 39 of them reported that SDF was now part of their curriculum, significantly different (*p* < 0.001) from 2016 (68% of schools). Significant changes (*p* < 0.001) were reported in the survey responses from 2016 to 2022, particularly in how SDF was being taught (97% teaching didactically and clinically vs. 48% in 2016), but were not necessarily a response to the COVID pandemic. Schools report teaching more indications for the use of SDF and using more specific protocols with more schools teaching arresting caries in permanent teeth (97% vs. 78% in 2016). Since 2016, the use and teaching of SDF have been increasingly adopted across U.S. dental schools, though there remains room for improvement in terms of consistent implementation, specific clinical protocols, and comprehensive training.

## Introduction

Silver Diamine Fluoride (SDF) is a Food & Drug Administration cleared agent for use as a low-cost topical agent for dentin hypersensitivity, available in the U.S since 2014. SDF can be used off-label for arresting carious lesions if the dental professional deems it appropriate. The use of SDF as a caries control technique has been around in other countries since 1969 (1). Inclusion of SDF in dental curriculum has been demonstrated to impact willingness and attitude towards SDF use (2, 3). Yet, although many dental educations programs are including SDF in the curriculum, implementation clinically in the education environment both in the US as well as abroad has been slow (2, 4, 5). In 2016, a survey on the use of Silver Diamine Fluoride (SDF) in predoctoral dental education programs in the U.S indicated that many schools were planning future inclusion of SDF in their schools' curricula (6).

Although U.S. schools are reportedly sluggish to adapt to new techniques, the momentum has slowly building for alternative evidence-based techniques (7). The COVID-19 pandemic was a driving force to implement several changes in dental education (8, 9). Dental schools across the US had to adapt their learning and clinical teaching, with most schools suspending clinical activities during the peak of the pandemic beyond emergency care and moving learning activities to online modalities (8–11). Faculty and administration were faced with balancing compliance with local government agencies and continuing to provide engaging education while keeping faculty, students, and patients safe (8). The pandemic impacted healthcare and the way dentistry was practiced worldwide. The aerosol-transmittable pathogen virus placed dental professionals at a high risk of exposure (12, 13). Recommendations have been made including but not limited to hand hygiene, antimicrobial agents, minimally invasive procedures, rubber dam, high-speed saliva ejectors, anti-retraction high-speed handpieces, and dental environment sanitation (12–14). with the experience acquired during the COVID-19 crisis, using non-surgical, non-aerosol-producing techniques could be invaluable. The aim of this study was to evaluate whether the use of SDF across the country and all CODA-accredited predoctoral (DDS/DMD) dental education programs has changed since 2016. The hypotheses were (1) The number of CODA accredited Predoctoral (DDS/DMD) dental education programs across the U.S that include SDF in their curriculum and the number of hours devoted to teaching SDF has increased since the 2016 survey (6) and (2) The COVID pandemic increased the current and future inclusion of SDF in the curriculum and its use in the clinic.

## Methods

The survey was modeled after a prior study titled “Teaching Silver Diamine Fluoride in U.S. Dental Schools” Predoctoral Curricula' (6). This 22-question survey (Qual­trics, Provo, UT, USA) was conducted by XXXX, IRB approved study #00002302.

### Survey development

Specifically, an IRB-approved recruitment script page and new questions pertaining to COVID were added (Table 1) and presented to 5 faculty members, using an IRB-approved script to evaluate the added questions for face and content validity. The selected faculty members attended departmental calibration sessions on SDF and were supervising its use in the clinical environment. The face validity questionnaire required the volunteer participants to evaluate the clarity, understandability, and comfort of the questions. Meanwhile, the content validity questionnaire required the volunteer participants to score from 0 to 5 (5 being very important) the importance of the questions about the survey and to answer if the specific question should be added to the survey or not. The responses were then used to determine if the questions should be added to the survey. On average the responses indicated that all questions should be added to the survey and no modifications indicated.

**Table 1:** SDF survey comparison.

	Survey year 2022	Survey year 2016	*P*-values
Is SDF included in the curriculum?			
Yes	39 out of 39 (100%)*	42 out of 62 (67.7%)	<0.001
No	0 out of 39 (0%)	20 out of 62 (32.3%)	
How is SDF taught?			
Didactically only	1 out of 38 (2.6%)	21 out of 42 (50%)	
Clinically only	0 out of 38 (0%)	1 out of 42 (2.4%)	
Both	37 out of 38 (97.4%)*	20 out of 42 (47.6%)	<0.001
Time devoted to teaching			
<1 h	0 out of 38 (0%)	13 out of 42 (32.5%)	
1–2 h	12 out of 38 (31.58%)	15 out of 42 (37.5%)	
2–5 h	19 out of 38 (50.0%)	10 out of 42 (25.0%)	
>5 h	7 out of 38 (18.42%)	2 out of 42 (5.0%)	
Are these indications taught for SDF?			
Dentin Hypersensitivity	26 out of 37 (70.3%)	29 out of 41 (70.1%)	
Caries prevention on primary teeth	16 out of 37 (43.2%)*	32 out of 41 (78.0%)	0.002
Arresting caries on primary teeth	36 out of 37 (97.3%)	40 out of 41 (97.6%)	
Caries prevention on root surfaces	20 out of 37 (54.1%)	23 out of 41 (56.1%)	
Arresting caries on root surfaces	36 out of 37 (97.3%)	34 out of 41 (82.9%)	
Caries prevention on permanent teeth	13 out of 37 (35.1%)	19 out of 41 (46.3%)	
Arresting caries on permanent teeth	36 out of 37 (97.3%)*	32 out of 41 (78.0%)	0.02
Was the inclusion of SDF due to direct effect of the COVID-19 Pandemic?		The following questions were added to 2022 survey in response to the pandemic	
No/No effect	37 out of 38 (97.4%)		
Yes	1 out of 38 (2.6%)		
Has the use of SDF in clinic changed since the beginning of COVID-19?			
No	32 out of 38 (84.2%)		
Yes	6 out of 38 (15.8%)		
How has the COVID-19 pandemic changed the didactic portion of SDF education?			
It has increased the didactic portion	1 out of 6 (16.7%)		
No change	5 out of 6 (83.3%)		
How has the COVID-19 pandemic changed the clinical portion of SDF education?			
It has increased the clinical portion	5 out of 6 (83.3%)		
No change	1 out of 6 (16.7%)		

* Significant results.

### Survey distribution

The survey was distributed, and data was collected between the dates of January 25th and February 25th, 2022. The contact information of potential participants was obtained from each school's website and included faculty that are responsible for the curriculum and curriculum-based decisions: deans, chairs, selected faculty members in restorative department, or equivalents. The recruitment email requested either a response to the survey or for the recipient to forward the email to the appropriate person in their school. All CODA (68) accredited (as of January 2022) predoctoral (DDS/DMD) dental education programs in the U.S. were invited to participate. After 2 weeks, a reminder email was sent out. As part of the survey the respondent had to select the school they represented, this still maintained the anonymity of the respondent but not the school.

### Statistical analyses

The survey included questions regarding SDF teaching at the institution and whether COVID influenced SDF inclusion in the curriculum. The survey determined the number of hours dedicated to teaching, specific protocols used for SDF, and/or future inclusion of SDF in the curriculum. No power estimates were conducted as it was a convenience sample (all US CODA accredited dental schools). Descriptive statistics were calculated. Associations between survey year and survey question were analyzed with the chi-square test. Fisher's exact test was used in the case of sparse cell counts. *P*-values less than 0.05 were considered statistically significant. All the data was analyzed using Stata 13.1 software (StataCorp LLC, College Station, TX, USA).

## Results

There were 60 responses recorded. This included multiple responses from the same institution and incomplete responses. Responses from the same institution were combined using Microsoft Excel. For multiple non-aligned responses received, data interpretation was performed as in the previous study (6). If conflicting responses were received, the negative response was ignored and the positive was used, this interpretation considered that different departments/divisions may be unaware of others adopting SDF and its use within the same institution. Thus, if any response from the same institution indicated that SDF was used, that response was included.

In total, at least one response was received from 39 (57%) of the 68 CODA-accredited predoctoral (DDS/DMD) dental education programs, 14 of them were private institutions, the others public institutions (list of schools provided as Supplementary Material). Thus, the responses represented 64.% of all public dental education programs in the US. Responses based on the ADEA regional structure included 6 schools from the Northeast, 12 from the Southeast, 11 from the Midwest, 4 from the Central region, and 7 from the Western region. One response indicated that SDF was taught, but all remaining responses were incomplete. Most other survey responses were complete.

Results are presented indicating how many responses were received for each question (Table 1). Most schools (97.4%; *n* = 37 out of *n* = 38) were teaching SDF both didactically and clinically over 2–5 h (50%; *n* = 19 out of *n* = 38). Depending on the institution the departmental names and structure vary, but the majority of SDF education is being done in the departments of cariology and comprehensive care, operative/restorative, pediatric, diagnostic, community health, primary care, prosthodontics, general dentistry, and public health. Arresting caries on primary teeth and permanent teeth and arresting root surface caries were the most predominant indications at 97.3% (*n* = 36 out of *n* = 37).

Most schools (84.2%; *n* = 32 out of *n* = 36) reported using specific protocols mainly for arresting caries on primary teeth, arresting root surface caries, and arresting caries on permanent teeth. Fewer schools reported having specific protocols for dentin hypersensitivity (34.3%; *n* = 12 out of *n* = 35), caries prevention on primary teeth (28.6%; *n* = 10 out of *n* = 35) and prevention of caries on permanent teeth (25.7%; *n* = 9 out of *n* = 35). Arresting carious lesions using multiple applications over several weeks was the most common approach (56.8%; *n* = 21 out of *n* = 37) followed by a single application (21.6%; *n* = 8 out of *n* = 37) and more than one application at a single appointment (8.1%; *n* = 3 out of *n* = 37). Some schools reported teaching more than one type of application protocol. One application only and multiple applications over several weeks are being taught by 8.1% (*n* = 3 out of *n* = 37) of schools. More than one application at a single appointment and multiple applications over several weeks are being taught by 5.41% (*n* = 2 out of *n* = 37).

The main type of follow-up after arresting caries was re-application of SDF on the arrested lesion (37.8%; *n* = 14 out of *n* = 37), restoring the lesions (27%; *n* = 10 out of *n* = 37) and observation only of arrested lesion (18.9%; *n* = 7 out of *n* = 37). Two of the respondents (5.4%; *n* = 2 out of *n* = 37) reported: observation only and re-application of SDF, observation only and restoration of the lesion, re-application of SDF, and restoring lesion as a follow-up.

Among the 18 institutions that teach re-application of silver diamine fluoride after initially arresting lesion with SDF, twice per year was the most common frequency (72.2%; *n* = 13 out of *n* = 18), followed by four times per year (11.1%; *n* = 2 out of *n* = 18), once per year (5.6%; *n* = 1 out of *n* = 18, while others (11.1%; *n* = 2 out of *n* = 18) reported that it depends on the severity and extent of lesion.

Overwhelmingly institutions reported that COVID had no impact on inclusion of SDF on the curriculum (97.4%; *n* = 37 out of *n* = 38) or on the use of SDF in the clinic (84.2%; *n* = 32 out of *n* = 38). Only one school reported an increase in the didactic portion of SDF education (16.7%; *n* = 1 out of *n* = 6), but most reported an increase in the clinical portion of SDF education (83.3%; *n* = 5 out of *n* = 6).

## Discussion

In the intervening 6 years between the administration of the previous survey (6) and the current survey, significant differences were found regarding the inclusion of SDF in the curriculum, how SDF was taught to dental students, indications for SDF use taught, and existing protocols for arresting caries in permanent teeth and arresting root caries as seen in Table 1. Additionally, most schools reported that COVID had no impact on how SDF is taught didactically and clinically (Table 1).

The number of schools that have existing protocols for both arresting caries in permanent teeth (*p* = 0.001) and arresting root caries (*p* < 0.001) has significantly increased. In 2016, an existing protocol for arresting caries in permanent teeth was reported by 50% (*n* = 12 out of *n* = 24), and an existing protocol for arresting root caries was reported 45.8% (*n* = 11 out of *n* = 24) (6). In comparison this survey reported that both protocols existed for 88.9% (*n* = 32 out of *n* = 36) of respondents.

The high inclusion rate (all 39 responding institutions) indicated a shift in the use of SDF and follows the suggestion of the previous survey study (6). The shift in inclusion is a promising move forward for U.S. Dental schools making SDF as part of their education and an available material/tool in the clinics and to the population they serve. There have been many reports on how different populations (15–20) can benefit from SDF, and training dental students is one step to increase use in clinical practice and acceptance (2, 3, 21). Graduating dental students have been shown to have a positive outlook towards the use of SDF and are inclined to use it in private practice (2). Currently there is still limited use of SDF in dentistry, but practitioners expect improved utilization of SDF (21, 22).

In comparison to the most popular SDF use indication from 2016 (arresting caries in primary teeth) (6), arresting caries in permanent teeth has become a widely accepted indication equivalent to arresting caries in primary teeth and arresting root caries. The addition of new indications could suggest further off-label use of SDF has become more acceptable in U.S. dental schools. The indications taught in dental schools for SDF use have shifted from caries prevention to caries arrest, aligning with ADA-published guidelines on SDF application (23). Further propagation and establishment of clear guidelines by existing authoritative institutions such as the ADA, could reinforce the current trend in U.S.-based dental schools and lead to overall higher use of SDF across institutional and private practice.

Recent reviews still show wide variability in SDF use protocols regarding indications, frequency, and follow-up (24–27). Improvements/changes are being made at institutional levels by expert panels with the help of students, faculty, and community health (28). Further indications are being considered for SDF, such as using SDF in non-esthetic areas for grossly open crown margins when conservation is indicated (29). However, these emerging applications lack robust evidence, underscoring the need for further research to establish best practices. Notably, U.S. dental schools have yet to adopt standardized protocols or provide evidence supporting these alternative uses of SDF, reflecting a gap in the implementation and dissemination of effective guidelines. Developing a comprehensive curriculum to support knowledge and clinical application of SDF is essential for advancing its use in dental practice.

This study indicates that SDF use and teaching of SDF has significantly increased in the US dental schools over the past 6 years. However, it also demonstrated that evidence-based clinical protocols must be developed and disseminated. Additionally, policymaking must catch up to currently available evidence, as there is a wide variability in insurance coverage and reimbursement as well as license limitations on its use.

## Limitations

The response rate to the survey was moderate (57%) and much lower than the previous survey (94% response rate). This may reflect the timing of the survey distribution in the calendar year. Because the survey was conducted from January 15th to February 15th, the holiday season prior to the survey could have affected the response rate. In addition, schools have been operating under COVID guidelines and the holiday season was a stressor resulting in higher cases and a busier time for educators to re-introduce students back into clinics. It has been reported that schools in the US have experienced higher financial and operational strains with budget cuts, revenue loss, and changes in faculty and staff (9, 10). Isolation of at-risk groups further reduced the already decreasing numbers of dental educators (30). The survey distribution method was via email, this could have been further improved by mailed surveys (31). Furthermore, since the data was de-identified it was not possible to compare if the schools that responded to the current survey were the ones that already had included SDF in the curriculum in the previous study or if the faculty completing the survey were the same faculty with the same or different affiliations. The majority of schools that responded (64.1%) were public schools and they represent 80% of all public dental education programs in the US. Based on the responses it was not possible to determine if the private schools that responded were any different than those that did not respond. It is notable how in the past 6 years SDF teaching and use has diffused across US dental schools and how interdepartmental inclusion has expanded ensuring exposure of dental students to SDF. COVID did not appear to impact to have an impact on the didactic component or clinical use, but for the few schools (*n* = 6) responding to this question it led to increase in clinical education on SDF. Again, this may indicate that the institutions that did respond had implemented inclusion of SDF in the curriculum prior to the COVID pandemic.

Ultimately, providing SDF as a caries management strategy can reduce expenditure (32), and Medicaid programs should consider reimbursement for its use in pediatric patients (22) and beyond. According to the AAPD as of 2021, Code D1354 was covered by 35 states (24). The use of SDF when combined with expanded practice dental hygienists can further lower the costs and expand the access to patients, especially in the underserved populations (33, 34). Expanded coverage of SDF by Medicaid and higher overall acceptance of SDF (35, 36) require dental professionals to advocate for it. However, it is important to consider that SDF should ideally be applied biannually to maximize its effectiveness, which represents an ongoing cost (37).

## Conclusion

Overall, an increasing trend in the inclusion of SDF in dental education was observed, with broad implications for population-level oral health. Its cost-effectiveness and potential long-term benefits may help patients retain their natural dentition longer, contributing to improved health outcomes.

## Data Availability

The raw data supporting the conclusions of this article will be made available by the authors, without undue reservation.
