# Supplementary material for: An updated look at the use of silver diamine fluoride in U.S. dental schools' predoctoral curriculum—a survey
Source: Front Dent Med. 2025 Jan 14;5:1466962. doi: 10.3389/fdmed.2024.1466962 (PMC11797966; doi:10.3389/fdmed.2024.1466962)
Supplement: Supplementary file 1 [file Datasheet1.docx]

**A.T. Still University Arizona School of Dentistry & Oral Health** - Mesa, Arizona (**Private**)

**California Northstate University College of Dental Medicine** - Elk Grove, California (**Private**)

**Case Western Reserve University School of Dental Medicine** - Cleveland, Ohio (**Private**)

**Creighton University School of Dentistry** - Omaha, Nebraska (**Private**)

**Harvard University School of Dental Medicine** - Boston, Massachusetts (**Private**)

**Herman Ostrow School of Dentistry of the University of Southern California** - Los Angeles, California (**Private**)

Indiana University School of Dentistry - Indianapolis, Indiana (Public)

**LECOM College of Dental Medicine** - Bradenton, Florida (**Private**)

**Meharry Medical College School of Dentistry** - Nashville, Tennessee (**Private**)

**New York University College of Dentistry** - New York City, New York (**Private**)

**Nova Southeastern University College of Dental Medicine** - Fort Lauderdale, Florida (**Private**)

Ohio State University College of Dentistry - Columbus, Ohio (Public)

Rutgers School of Dental Medicine - Newark, New Jersey (Public)

Southern Illinois University School of Dental Medicine - Alton, Illinois (Public)

Texas A&M University College of Dentistry - Dallas, Texas (Public)

Texas Tech University Health Sciences Center El Paso - El Paso, Texas (Public)

The University of Texas School of Dentistry at Houston - Houston, Texas (Public)

University of Colorado Denver School of Dental Medicine - Aurora, Colorado (Public)

**University of Detroit Mercy School of Dentistry** - Detroit, Michigan (**Private**)

University of Florida College of Dentistry - Gainesville, Florida (Public)

University of Illinois at Chicago College of Dentistry - Chicago, Illinois (Public)

University of Iowa College of Dentistry - Iowa City, Iowa (Public)

University of Kentucky College of Dentistry - Lexington, Kentucky (Public)

University of Louisville School of Dentistry - Louisville, Kentucky (Public)

University of Michigan School of Dentistry - Ann Arbor, Michigan (Public)

University of Minnesota School of Dentistry - Minneapolis, Minnesota (Public)

University of Mississippi School of Dentistry - Jackson, Mississippi (Public)

University of Missouri-Kansas City School of Dentistry - Kansas City, Missouri (Public)

**University of New England College of Dental Medicine** - Biddeford, Maine (**Private**)

University of North Carolina at Chapel Hill Adams School of Dentistry - Chapel Hill, North Carolina (Public)

University of Oklahoma College of Dentistry - Oklahoma City, Oklahoma (Public)

**University of Pennsylvania School of Dental Medicine** - Philadelphia, Pennsylvania (**Private**)

University of Pittsburgh School of Dental Medicine - Pittsburgh, Pennsylvania (Public)

University of Utah School of Dentistry - Salt Lake City, Utah (Public)

University of Washington School of Dentistry - Seattle, Washington (Public)

University of Alabama at Birmingham School of Dentistry - Birmingham, Alabama (Public)

VCU School of Dentistry - Richmond, Virginia (Public)

West Virginia University School of Dentistry - Morgantown, West Virginia (Public)

**Western University of Health Sciences College of Dental Medicine** - Pomona, California (**Private**)
